# Methane Flux Responses to Warming and Inundation in the Qinghai Lake Littoral Wetland

**DOI:** 10.3390/biology15110840

**Published:** 2026-05-27

**Authors:** Hairui Zhao, Ziwei Yang, Yanfen Yang, Mingzhu Cao, Yuyu Ma, Chen Chen, Shuchang Zhu, Kelong Chen

**Affiliations:** 1 School of Life Sciences, Qinghai Normal University, Xining 810008, China; 18697237052@163.com; 2 Key Laboratory of Natural Geography and Environmental Processes of Qinghai Province, Xining 810008, China; 15756789182@163.com (Z.Y.); 19143266764@163.com (Y.Y.); cmz6154@163.com (M.C.); myy6253@163.com (Y.M.); ccreus1nj@163.com (C.C.); zhushuchang@163.com (S.Z.); 3 Key Laboratory of Qinghai-Tibet Plateau Surface Process and Ecological Conservation, Ministry of Education, Qinghai Normal University, Xining 810008, China; 4 School of Geographical Sciences, Qinghai Normal University, Xining 810008, China

**Keywords:** climate change, Qinghai Lake Basin, littoral wetland, greenhouse gases, water level rise

## Abstract

This study investigated the interactive effects of warming and inundation on methane (CH_4_) fluxes and soil physicochemical mechanisms in the littoral wetland of Qinghai Lake. Soil samples were collected from the Bird Island littoral wetland. Eight treatments were established: natural control (CK), different inundation depths (S0, S10, S20), warming alone (ZWCK), and warming combined with inundation (ZW0, ZW10, ZW20). CH_4_ fluxes were measured over one year using an ABB LGR analyzer. Principal component analysis (PCA) and Mantel tests were used to identify environmental drivers. The main findings are as follows: (1) Under different water level treatments, CH_4_ fluxes showed a unimodal seasonal pattern, peaking in autumn. Warming and the interactive treatments shifted the emission pattern to bimodal or multimodal and significantly increased emission intensity. The warming-alone group had the highest annual emission, with anomalously high winter emission (47.683 μg·m^−2^·h^−1^). Under the ZW20 treatment, emissions were synergistically enhanced in summer and autumn but turned to suppression in winter. (2) PCA showed that the carbon nitrogen pool (70.5%) and the salinity pH gradient (14.9%) were the main drivers of soil variation. The interactive effects on carbon-nitrogen dynamics shifted with season: warming promoted accumulation in spring; warming with shallow inundation retained carbon-nitrogen in summer, but deep inundation caused loss; warming with deep inundation formed a nutrient center in autumn; inundation dominated accumulation in winter, while warming increased loss. (3) Mantel tests showed that carbon-nitrogen components were highly correlated across seasons, but were strongly and positively correlated with CH_4_ flux only in autumn (Mantel’s r ≥ 0.4, *p* < 0.05), indicating autumn as the key window. These findings provide important insights into carbon cycling processes and regulatory mechanisms of alpine wetlands under future climate change scenarios.

## Simple Summary

Global warming and altered flooding patterns significantly affect methane emissions from alpine wetlands. This study conducted a one-year experiment in the Qinghai Lake littoral wetland with warming and different inundation depths to explore their interactive effects on methane fluxes. The results showed that under flooding alone, methane emissions peaked in autumn. Warming not only changed the seasonal pattern of emissions but also caused anomalously high emissions in winter. Warming combined with deep flooding enhanced emissions in summer and autumn but suppressed them in winter. Soil carbon and nitrogen components were closely related to methane emissions, but this relationship was only significant in autumn. Overall, the interaction between warming and flooding on methane emissions is strongly season-specific, and autumn is a key window for carbon-nitrogen coupling driving methane emissions. Future climate warming and hydrological extremes may further increase the risk of methane release from littoral wetlands.

## 1. Introduction

Global climate change is mainly characterized by rising temperatures and altered precipitation patterns. According to the IPCC Sixth Assessment Report, global surface temperature increased by 1.09 °C from 2011 to 2020 compared to pre-industrial levels, and may exceed the 1.5 °C threshold in the future [1]. This warming trend is particularly significant on the Qinghai-Tibet Plateau, where the warming rate is about twice the global average [2]. In recent years, the Qinghai Lake Basin has shown a clear trend of warming and wetting [3]. Over the past 50 years, the annual maximum and minimum temperatures have increased by 1.53 °C and 1.67 °C, respectively [4]. From 2005 to 2020, the lake water level had risen by more than 3 m [5]. These rapid climate-hydrological changes are profoundly affecting the carbon cycling processes of the Qinghai Lake littoral wetland ecosystem.

Wetlands are important ecological interfaces between land and water. Although they cover only 3.7% of the global land area [6], they store 20–30% of the global soil carbon pool and are major sources of atmospheric methane (CH_4_) [7]. Because the global warming potential of CH_4_ is about 28–34 times that of CO_2_ on a century-scale [1], even small changes in CH_4_ flux can have significant feedback effects on the climate system. Under the background of global warming and intensified hydrological changes, CH_4_ emission processes in wetlands show considerable uncertainty. Previous studies have shown that rising temperatures can directly promote CH_4_ production by accelerating organic matter decomposition. Changes in inundation patterns indirectly regulate CH_4_ production and oxidation by modifying soil redox conditions, pH, organic carbon availability, and other physicochemical factors [8]. Temperature and water availability are two key environmental factors regulating CH_4_ emissions in wetlands. They often co-occur and may produce additive, synergistic, or antagonistic effects [9,10,11]. Temperature changes alter soil hydrothermal conditions, affecting substrate utilization by methanogens, thus modifying the temperature sensitivity of CH_4_ emissions and leading to emission differences [12]. Under soil water limitation, warming may reduce CH_4_ emissions [13] or have no significant effect [14]. This difference arises from variations in wetland type and temporal scale, which alter the intensity and mechanisms of the factors [15,16]. Therefore, this study used soils from the Bird Island littoral wetland of Qinghai Lake to investigate the interactive effects of warming and inundation on CH_4_ fluxes, providing a scientific basis for understanding the carbon cycling processes of the Qinghai Lake littoral wetland and their feedback to climate change.

## 2. Materials and Methods

### 2.1. Study Area

**Figure 1:**
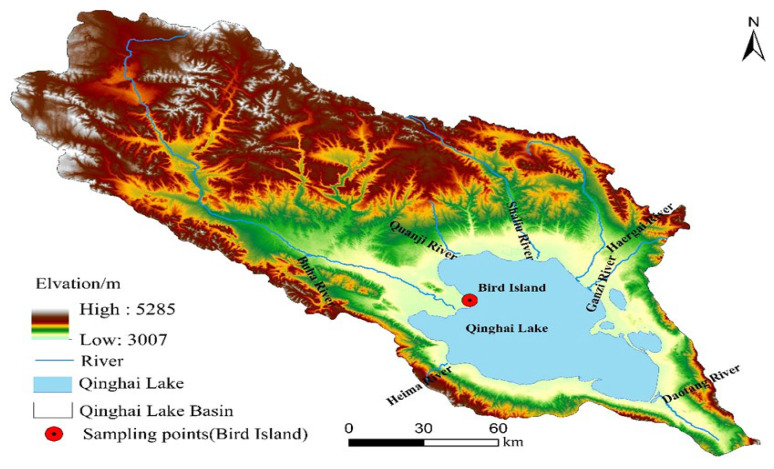
Overview of the study area.

The Bird Island Station is located in the northwestern part of Qinghai Lake (36°57′ N–37°04′ N, 99°44′ E–99°54′ E) (Figure 1), at an altitude of 3194–322 m, with a total area of about 600 km^2^. The study area is a plateau littoral wetland ecosystem. The terrain is higher in the northwest and lower in the southeast. Influenced by the East Asian monsoon and the westerly winds of the Qinghai Tibet Plateau, the climate is semi-arid and alpine. The annual mean air temperature is −0.7 °C, with the warmest month (July) averaging 12.4 °C and the coldest month averaging −12.7 °C. Excessively low temperatures lead to the formation of a frozen layer, which reduces soil permeability, impedes gas diffusion, and consequently affects methane emissions [17,18]. The mean annual precipitation is 420 mm, and evaporation is 3.8 times the precipitation. The average number of strong wind days per year is 48, and can reach up to 78 days. Vegetation height ranges from 30 to 40 cm, and vegetation cover exceeds 60%. The main plant species include *Leymus secalinus*, *Artemisia frigida*, and *Poa alpigena*. The soil type is sandy loam rich in gravel, formed by the weathering of Triassic or Permian gneiss and coastal sediments. Since 2004, the water level of Qinghai Lake has been continuously rising. By 2018, the littoral wetland had expanded by 20–500 m compared to 2014, and the inundated area increased by 21.86 km^2^ [19].

### 2.2. Data and Methods

**Figure 2:**
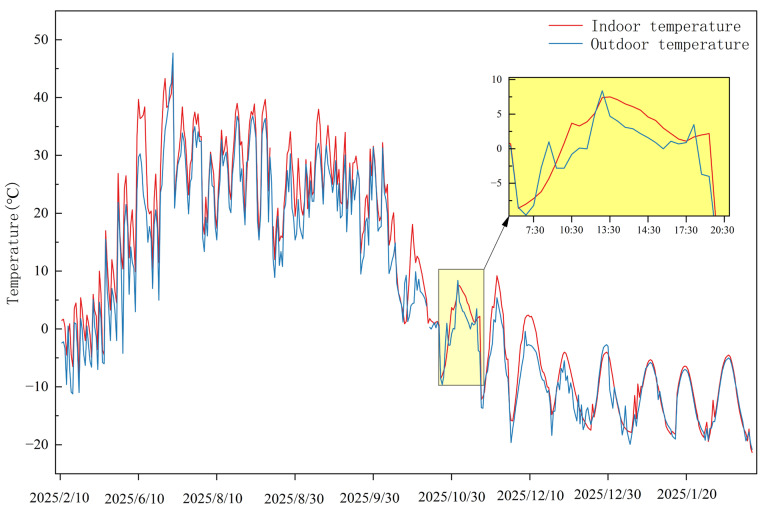
Trends of indoor and outdoor temperatures.

In August 2024, soil rings (25 cm in diameter, 50 cm in height) were vertically driven into the soil at the Bird Island littoral wetland of Qinghai Lake to collect soil from the 0–35 cm layer. The collected soil was layered into acrylic tubes (inner diameter 24 cm, outer diameter 25 cm, height 50 cm). The bottom of each tube was sealed, and holes (1.6 cm in diameter) were drilled on the side at intervals of 10 cm. The tubes were wrapped with a light shielding film (1.8 cm thick) to prevent light from affecting the soil inside. Experimental devices were set up to simulate warming and lake level rise (inundation). The treatments were as follows: natural control (CK), inundation at 0 cm above the soil surface (S0), inundation at 10 cm (S10), inundation at 20 cm (S20), warming alone (ZWCK), and the interactive treatments of warming with inundation at 0 cm (ZW0), 10 cm (ZW10), and 20 cm (ZW20). Each treatment had three replicates. The water used for inundation during the experiment was collected in situ from the lake. The warming experiment was set up in August 2024, following the structure of a greenhouse commonly used in the Qinghai Lake Basin. The top was made of polycarbonate hollow sheets with 89.5% light transmittance, and the sides were enclosed with color steel sheets to maintain temperature. Holes were made in the color steel sheets to ensure that the average temperature inside the chamber was about 4 °C higher than outside (Figure 2), Temperature monitoring was performed using a temperature data logger (Onset HOBO U23-001, Beijing Jiuzhou Shengxin Technology Co., Ltd., Beijing, China).

After six months of incubation to allow the soil columns to stabilize, CH_4_ fluxes were measured in spring, summer, autumn, and winter starting from March 2025 using an ABB LGR (GGA-911, ABB Inc., Saint-Laurent, QC, Canada) greenhouse gas analyzer. Meanwhile, soil was sampled once in the middle of each quarter to determine seasonal changes in soil pH (PHS-25, Shanghai Instruments and Electrical Science Co., Ltd., Shanghai, China), electrical conductivity (EC) (DDS-307, Shanghai Instruments and Electrical Science Co., Ltd., Shanghai, China), total carbon (TC) (ECS4024, LICA United Technology Co., Ltd., Beijing, China), total nitrogen (TN) (ECS4024, LICA United Technology Co., Ltd., Beijing, China), total organic carbon (TOC) (was determined by the potassium dichromate-external heating oxidation method), total inorganic carbon (TIC) (was calculated by the subtraction method, TIC = TC–TOC), and microbial biomass carbon (MBC) (was determined by the chloroform fumigation-potassium sulfate extraction method). Mantel tests and Pearson correlation analysis were then used to identify the dominant factors controlling CH_4_ emissions under the interaction of warming and inundation and their seasonal differences, and to clarify the regulatory role of seasonal dynamics of soil physicochemical factors on CH_4_ emissions. Gas samples were collected every 10 days. At each sampling event, gas samples were taken at 30 min intervals. After obtaining the data, the CH_4_ flux was calculated using the concentrations at 0, 10, and 30 min according to the following formula [20].

(1) $$F=\rho \times \frac{V}{A}\times \frac{P}{P_{0}}\times \frac{T_{0}}{T}\times \frac{dC_{t}}{dt}$$

where *F* is the greenhouse gas flux (mg·m^−2^·h^−1^); *ρ* is the gas density under standard conditions (g·L^−1^); *V* is the volume of the static chamber (m^3^); *A* is the area covered by the static chamber (m^2^); *P* is the air pressure at the sampling site (hPa); *P*_0_ is the standard atmospheric pressure (hPa); *T*_0_ is the absolute air temperature under standard conditions (K); *T* is the absolute air temperature inside the chamber at the time of sampling (K); *dC_t_*/*dt* is the rate of change in the measured gas concentration inside the chamber over time.

### 2.3. Statistical Analysis

Origin 2025b software was used to generate figures and to analyze differences in greenhouse gas fluxes among different sampling times and treatments. R language (R 4.4.3) was used to perform principal component analysis (PCA) of soil physicochemical indicators across different seasons, to create heatmaps of these indicators under different treatments, and to conduct Mantel tests and correlation analyses between seasonal CH_4_ fluxes and soil physicochemical properties. In the Mantel test, the Euclidean distance was used for the environmental factor matrix, while the Bray–Curtis distance was applied to the CH_4_ flux matrix. Seasonal variations and differences in soil physicochemical properties among treatments were analyzed. Pearson correlation analysis was used to examine the covariations among soil physicochemical properties. Mantel tests were employed to assess the significance and effect size of the associations between environmental factors and CH_4_ fluxes.

## 3. Results

### 3.1. Monthly and Seasonal CH_4_ Flux Characteristics Under Warming and Different Inundation Gradients

Under the inundation treatments, CH_4_ fluxes in all treatments showed a typical unimodal seasonal pattern, generally increasing first and then decreasing. The peak emissions occurred from July to September (autumn). Except for the S0 treatment, all other treatments reached their highest peaks in August: 59.430 μg·m^−2^·h^−1^ (CK), 52.844 μg·m^−2^·h^−1^ (S10), and 53.843 μg·m^−2^·h^−1^ (S20). The S0 treatment peaked in October, reaching 62.231 μg·m^−2^·h^−1^. Across the entire observation period, the annual CH_4_ fluxes under different inundation gradients followed the order S0 > S20 > S10 > CK. Weak CH_4_ uptake was observed from January to March and in December, while emissions occurred during the rest of the year. This indicates that inundation promotes CH_4_ emissions.

The interactive treatments of warming and inundation significantly altered the dynamics of CH_4_ fluxes, showing bimodal or multimodal patterns with greatly increased peak values. In terms of annual total emissions, the order among warming treatments was ZWCK > ZW20 > ZW10 > ZW0. This suggests that the interaction between warming and inundation on CH_4_ emissions is not simply synergistic or antagonistic. Although deep inundation combined with warming showed synergistic enhancement in summer and autumn, it was inhibited in winter. The warming alone treatment (ZWCK) had the highest annual total emission, indicating that the direction and intensity of the warming effect strongly depend on water conditions and seasonal rhythms.

**Figure 3:**
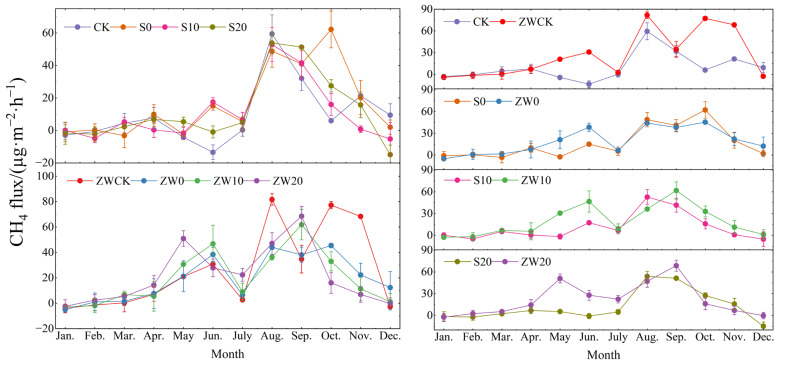
Monthly CH_4_ emission patterns.

CH_4_ fluxes under the warming and interactive treatments were generally higher than those under inundation alone. The ZWCK group reached its annual peak in August (81.710 μg·m^−2^·h^−1^), which was 37.49% higher than that of the CK group. Warming also extended the high emission period from summer into autumn, indicating that warming directly promotes CH_4_ emissions (Figure 3).

**Figure 4:**
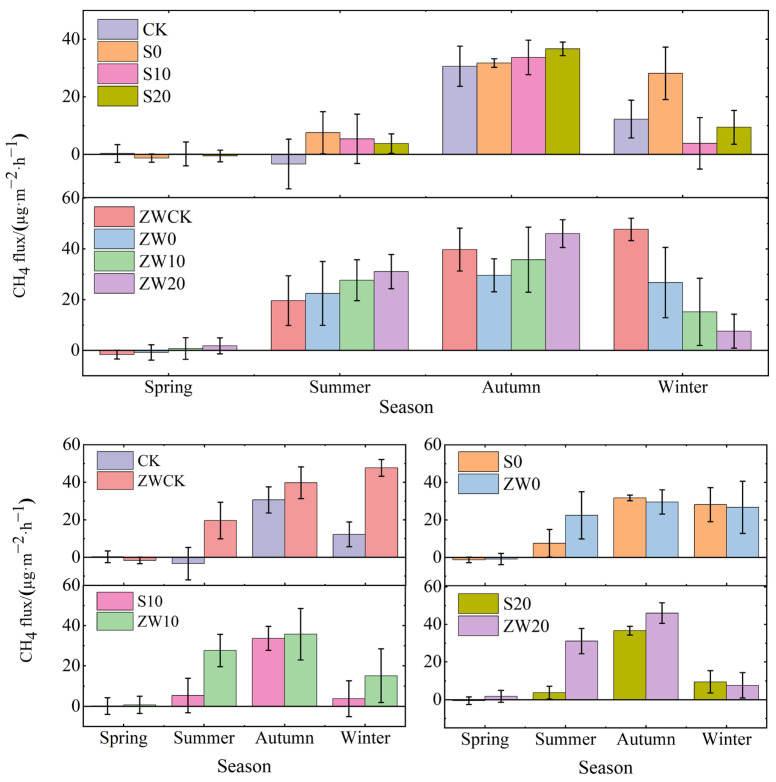
Seasonal CH_4_ emission patterns.

On a seasonal scale, autumn was the main season for CH_4_ emissions under inundation treatments (peak values ranging from 30.595 to 36.652 μg·m^−2^·h^−1^). In contrast, warming completely changed the seasonal distribution pattern: the ZWCK group had the highest emissions in winter (47.683 μg·m^−2^·h^−1^), showing a counter-seasonal characteristic. Warming combined with deep inundation (ZW20) produced the strongest synergistic enhancement effect in autumn (emission of approximately 46.029 μg·m^−2^·h^−1^), but showed an inhibitory effect in winter (Figure 4). Overall, CH_4_ emissions had clear emission windows in summer and autumn, when high temperatures and stable inundation conditions overlapped, driving large CH_4_ production. Warming and inundation each independently promoted emissions, but their combined synergistic effect was much greater than the sum of their individual effects, and this interaction was most significant in summer and autumn. The ZW20 treatment consistently maintained the highest emission levels across all seasons, suggesting that the combination of high temperature and deep inundation may be a key concern under future climate warming, accompanied by extreme precipitation scenarios.

### 3.2. Soil Physicochemical Properties Under Warming and Different Inundation Gradients

**Figure 5:**
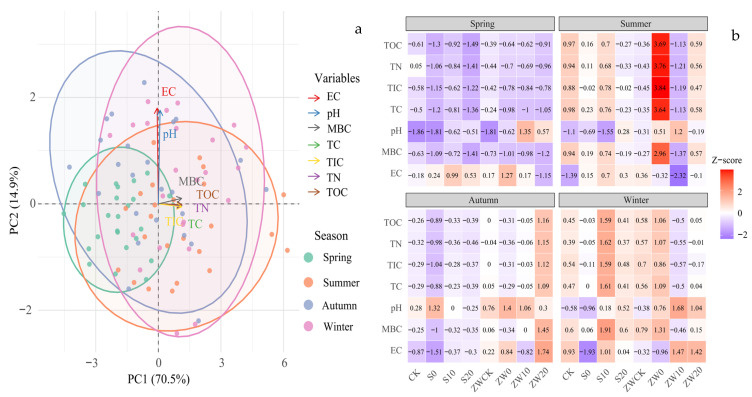
Principal component analysis (PCA) of soil physicochemical indicators in different seasons (**a**) and responses of soil physicochemical properties to warming and inundation (**b**).

To reveal the seasonal variation characteristics of soil physicochemical properties and their dominant drivers, principal component analysis (PCA) was performed on soil physicochemical indicators (pH, TOC, TN, TIC, MBC, etc.) under different treatments across seasons. The results are shown in Figure 5a.

PCA showed that the first two principal components cumulatively explained 85.4% of the total variation in soil physicochemical properties. The first principal component (PC1) explained 70.5% of the variation, and the second principal component (PC2) explained 14.9%. PC1 was positively correlated with TC, TIC, TN, TOC, and MBC, reflecting the gradient of soil carbon nitrogen components. PC2 was mainly influenced by pH and EC, and is positively correlated with both, reflecting differences in soil acidity/alkalinity and salinity.

Samples from different seasons showed distinct separation in the ordination space, indicating that seasonal changes significantly affect the overall characteristics of soil physicochemical properties. Spring samples were mainly distributed in the negative region of PC1, suggesting that carbon- and nitrogen-related indicators were generally low in this season. Autumn and winter samples were mostly distributed in the positive region of PC1, indicating that TC, TIC, TN, and TOC were relatively higher. Summer samples showed the widest distribution, suggesting greater variation in soil physicochemical properties during this season and more pronounced differences among treatments (Figure 5a).

To further compare seasonal differences among treatments, a heatmap (Figure 5b) and a difference analysis (Table 1) were conducted for soil physicochemical properties across the four seasons. The results showed that warming, inundation, and their interactive treatments significantly affected soil physicochemical indicators in each season.

In spring, the scores of TOC, TN, TIC, TC, and MBC were generally negative across treatments. Under the same inundation conditions, the carbon-nitrogen component scores of the warming treatments were generally higher than those of the corresponding non-warming treatments. Meanwhile, pH was lower in the CK and ZWCK treatments but higher in ZW10 and ZW20. EC was relatively higher in the S10 and ZW0 treatments.

In summer, the ZW0 treatment had generally higher scores for TOC, TN, TIC, TC, and MBC, while most indicators scored lower in the ZW10 treatment. pH was relatively lower in CK and S10 but higher in ZW10. EC was highest in S10 and lowest in ZW10, indicating that soil physicochemical properties varied significantly among warming treatments under different inundation depths in summer.

In autumn, the ZW20 treatment had generally higher scores for TOC, TN, TIC, TC, MBC, and EC, while most indicators scored lower in the S0 treatment. pH was higher in S0, ZW0, and ZW10, and slightly lower in S20, indicating a clear differentiation between the deep inundation plus warming treatment and the other treatments in autumn.

In winter, the S10 treatment had generally higher scores for TOC, TN, TIC, TC, MBC, and EC, while EC was lower in the S0 treatment. pH was higher in ZW10 and ZW20, and lower in CK and ZWCK. However, TOC and TN scores were relatively lower in the ZW10 treatment, indicating that carbon-nitrogen components and acidity-salinity characteristics varied significantly among treatments in winter.

The temporal variation in soil physicochemical properties was jointly characterized by two gradients: the carbon-nitrogen component (PC1, 70.5%) and the acidity-salinity gradient (PC2, 14.9%). The direction and magnitude of the effects of warming and inundation on soil physicochemical properties varied significantly across seasons, showing strong seasonal dependence.

### 3.3. Mantel Test and Correlation: Seasonal CH_4_ Fluxes vs. Soil Properties

To analyze the relationships between soil physicochemical properties and CH_4_ fluxes across different seasons, Pearson correlation analysis was used to examine the correlations among soil physicochemical factors based on soil samples collected in spring, summer, autumn, and winter. Mantel tests were then employed to assess the significance and strength of the associations between environmental factors and CH_4_ fluxes.

The Pearson correlation results showed that, in all seasons, soil carbon nitrogen components (including TC, TN, TOC, TIC, and MBC) were positively correlated with each other. In spring, the correlation coefficients among carbon nitrogen components ranged from r = 0.73 to 0.92, and increased to r = 0.98–1.00 in summer, autumn, and winter, indicating strong consistency among these indicators across seasons. Meanwhile, the relationship between EC and pH showed seasonal differences: they were negatively correlated in summer (r = −0.50) but positively correlated in winter (r = 0.57).

The Mantel test results revealed significant seasonal differences in the correlations between environmental factors and CH_4_ fluxes. In spring, summer, and winter, no environmental factor showed a significant Mantel correlation with CH_4_ flux (*p* ≥ 0.05). Specifically, carbon nitrogen components tended to show non-significant negative correlations with CH_4_ flux in spring and winter (|Mantel’s r| = 0.2–0.4), and a non-significant weak positive correlation in summer (|Mantel’s r| < 0.2). In contrast, in autumn, TC, TN, TOC, TIC, and MBC were all significantly positively correlated with CH_4_ flux (Mantel’s r ≥ 0.4, *p* = 0.01–0.05), while EC and pH still showed no significant correlations (*p* ≥ 0.05, |Mantel’s r| < 0.2).

**Figure 6:**
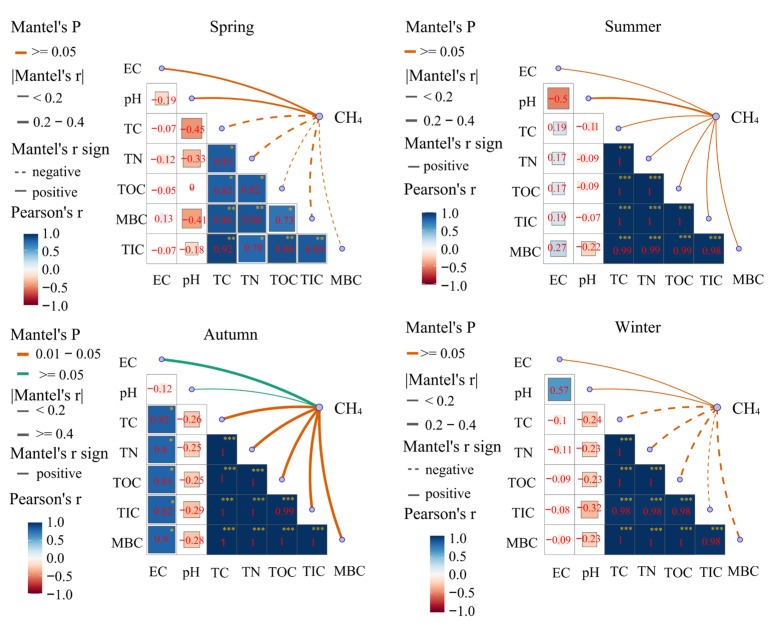
Mantel test and correlation analysis between seasonal CH_4_ fluxes and soil physicochemical properties. Note: *, **, and *** indicate *p* < 0.05, *p* < 0.01, and *p* < 0.001, respectively.

Overall, soil carbon and nitrogen components were strongly correlated with each other across all seasons, but were significantly positively correlated with CH_4_ flux only in autumn. This indicates that the link between soil carbon, nitrogen components, and CH_4_ emissions is closer in autumn, showing a clear seasonal pattern (Figure 6).

## 4. Discussion

### 4.1. Effects of Warming and Inundation on CH_4_ Fluxes

Changes in environmental factors and hydrological conditions have direct or indirect effects on soil CH_4_ fluxes [21]. Temperature and water availability are important drivers of material cycling and energy flow in ecosystems, and are also key environmental variables affecting CH_4_ production, oxidation, and transport in wetlands [22]. Zhu et al. also pointed out that water level is a core driver regulating CH_4_ emissions in wetlands [23], but its direction and intensity are influenced by environmental factors such as temperature and carbon-nitrogen substrates. The present study found that the interaction between warming and inundation on CH_4_ emissions in the Qinghai Lake littoral wetland has obvious interactive characteristics and shows strong seasonal dependence.

Under the inundation gradient, CH_4_ fluxes in all treatments generally followed a unimodal seasonal pattern, with annual CH_4_ emissions following the order S0 > S20 > S10 > CK. Monthly dynamics showed that before October, CH_4_ fluxes generally increased with inundation depth, consistent with the finding of Mander et al. that higher water levels lead to higher CH_4_ emissions [24]. This is likely because inundation enhances soil anaerobic conditions and promotes methanogenesis. The unusual peak in the S0 treatment in October may be due to decreased methane oxidation activity as temperatures dropped. Studies have shown that temperature affects methane oxidation by influencing the abundance and community structure of methane-oxidizing bacteria [25]. At the same time, excessively deep water may create a CH_4_ oxidation zone, reducing CH_4_ emissions at higher water levels [26], ultimately resulting in the S0 treatment having the highest annual CH_4_ flux.

Warming significantly changed the seasonal distribution pattern of CH_4_ emissions. The ZWCK treatment showed the highest emission in winter (47.683 μg·m^−2^·h^−1^), indicating that warming may enhance CH_4_ emission processes in the non-growing season. This result is consistent with the conclusion of Liu et al. on the Qinghai Tibet Plateau that non-growing season CH_4_ exchange is more responsive to warming [27]. The interaction between warming and inundation showed strong seasonal dependence, with a synergistic effect in summer and autumn. The ZW20 treatment (warming and deep inundation) had the highest emissions (approximately 46.029 μg·m^−2^·h^−1^), consistent with the synergistic mechanism proposed by He et al. that high temperature promotes methanogenesis and high water level maintains anaerobic conditions [28]. Here is the English translation of the sentence: However, the lower CH_4_ emission in the ZW20 treatment compared to ZWCK during winter is consistent with previous observations of CH_4_ emission suppression during ice-covered periods in alpine wetlands [18], suggesting that ice formation may have hindered gas diffusion pathways.

In terms of annual total emissions, the order among warming treatments was ZWCK > ZW20 > ZW10 > ZW0, indicating that the interaction between warming and inundation is not fixed as synergistic enhancement, but is jointly regulated by seasonal conditions and water depth. High temperature and deep inundation in summer and autumn favor increased CH_4_ emissions, while winter warming may extend the seasonal duration of CH_4_ emissions from wetlands. This suggests that under future climate warming and hydrological pattern changes, CH_4_ emission processes in alpine littoral wetlands may show stronger temporal heterogeneity, which should be taken into account in regional wetland carbon cycle assessments.

### 4.2. Effects of Warming and Inundation on Soil Physicochemical Properties

This study reveals that the interaction between warming and inundation on soil carbon and nitrogen dynamics in wetlands shows significant seasonal patterns. Principal component analysis indicated that soil property variation is mainly driven by two major gradients: the carbon nitrogen pool (PC1) and the salinity pH gradient (PC2). This is consistent with the consensus in wetland research that carbon, nitrogen cycling, and salinity are key factors [29].

The seasonal patterns are as follows. In spring, carbon nitrogen indicators were generally low, but warming increased soil carbon nitrogen components. This is consistent with the findings of Zou et al. [30]. Warming may also enhance microbial activity during low temperature periods, promote plant litter decomposition, and increase soil carbon and nitrogen components [31]. In summer, warming combined with shallow inundation (0 cm) synergistically promoted carbon nitrogen retention and microbial biomass growth. However, when the inundation depth increased to 10 cm, carbon nitrogen loss occurred, indicating that excessive deep inundation under high temperatures may induce strong anaerobic conditions and alter carbon cycling pathways. Related studies have also shown that while increased inundation inhibits aerobic mineralization of soil organic carbon, it promotes anaerobic mineralization of sediments [32]. In autumn, the dominant treatment shifted to warming combined with deep inundation (ZW20), suggesting that warming [33] and inundation [34] can form nutrient accumulation centers, which is consistent with the results of the present study. In winter, the pattern reversed fundamentally: S10 became the dominant treatment for carbon nitrogen accumulation, while warming exacerbated carbon nitrogen loss, indicating that winter warming may affect soil carbon nitrogen decomposition by altering microbial activity [25].

In summary, the direction of the interactive effects of warming and inundation strongly depends on seasonal water temperature combinations, ranging from synergistic promotion to reversal inhibition. This emphasizes that when assessing the impacts of climate change on wetland carbon sink functions, such dynamic seasonal mechanisms must be taken into account.

### 4.3. Effects of Changes in Soil Physicochemical Properties on CH_4_ Fluxes

This study found that carbon nitrogen components (TC, TN, TOC, TIC, and MBC) were highly correlated with each other across all seasons, and showed a strongly significant positive correlation with CH_4_ flux only in autumn. This result is consistent with the understanding that wetland methane emissions are co-regulated by organic carbon and nitrogen [35]. Related studies have also found that TC and TN can affect microbial diversity and community structure, thereby influencing carbon cycling processes [36]. The coupling of carbon and nitrogen components was stronger in the warm seasons (summer and autumn), possibly because high temperatures intensify microbial activity, promoting the simultaneous transformation of organic matter and nitrogen mineralization, thus providing more abundant substrates for methanogenesis [37].

It is noteworthy that the driving effects of environmental factors on CH_4_ fluxes showed strong seasonal specificity. No significant correlations were found in spring, summer, or winter, and only in autumn did carbon-nitrogen components show a strong positive correlation with CH_4_ flux. This is consistent with many studies indicating that the main controlling factors of wetland CH_4_ emissions vary seasonally [15,16]. Autumn is a key window for CH_4_ emissions, followed by summer. On the one hand, during the growing season, active plant growth and metabolism provide abundant substrates for methanogens. On the other hand, the higher temperatures in summer and autumn create favorable conditions for methanogens and other related microorganisms, promoting CH_4_ fluxes, while the low temperatures in spring and winter may inhibit microbial activity and thus suppress CH_4_ production and emission [38]. In addition, the seasonal reversal of the relationship between electrical conductivity (EC) and pH (negative correlation in summer, positive correlation in winter) suggests a seasonal dynamic in the interaction between salinity and acid-base balance. High temperature and high evaporation in summer may lead to salt accumulation and changes in ion composition, thereby affecting pH. Yang et al. also pointed out that higher salt concentrations in soil solution can affect soil acid-base balance [39]. This reversal may indirectly modulate the seasonal pattern of CH_4_ emission responses to substrates by influencing methanogen community structure or activity.

In summary, this study highlights that the carbon nitrogen coupling driving wetland CH_4_ emissions has a significant seasonal window effect, with autumn being the key period. This provides an important basis for predicting the responses of wetland methane emissions to future climate change (e.g., warming, hydrological pattern changes).

## 5. Conclusions

This study revealed the seasonal regulation mechanisms of the interactive effects of warming and inundation on CH_4_ emissions in the lakeside wetland of Qinghai Lake. The results showed that warming shifted CH_4_ emissions from a unimodal pattern (peak in autumn) to a bimodal/multimodal pattern, significantly increasing the total annual emissions. Among all treatments, the warming-alone treatment exhibited a counter-seasonal high emission in winter, while the combination of warming and deep inundation showed synergistic enhancement in summer and autumn but turned to suppression in winter. Principal component analysis indicated that soil physicochemical variations were primarily driven by the carbon-nitrogen component (70.5%) and the acidity-salinity gradient (14.9%). Although carbon-nitrogen components were highly correlated across all seasons, a strong positive correlation with CH_4_ flux occurred only in autumn. In summary, the effects of warming and inundation on CH_4_ emissions in the Qinghai Lake lakeside wetland are distinctly season-specific, with autumn being the critical window during which carbon-nitrogen components drive CH_4_ emissions. Future research should integrate long-term water table monitoring and microbial functional assays (e.g., methanogenesis and methane oxidation potentials) to better constrain the mechanistic pathways underlying greenhouse gas responses. Moreover, long-term in situ observations and multi-site comparative studies across the Qinghai–Tibet Plateau will be essential for improving model predictions of alpine wetland feedbacks to climate change.

## Figures and Tables

**Table 1:** Soil physicochemical properties under different treatments across seasons.

Season	Treatment	EC (μs/cm)	pH	TC (g/kg)	TN (g/kg)	TOC (g/kg)	TIC (g/kg)	MBC (mg/kg)
Spring	CK	1345.11 ± 5.09 d	8.07 ± 0.02 d	11.8 ± 1.52 b	1.04 ± 0.23 a	8.96 ± 1.75 ab	2.59 ± 0.31 ab	311.88 ± 1.46 a
	S0	1452.71 ± 2.84 c	8.08 ± 0.02 d	8.7 ± 0.84 cd	0.77 ± 0.1 bc	7.57 ± 1.1 ab	1.84 ± 0.11 cd	246.91 ± 3.46 d
	S10	1645.58 ± 47.59 a	8.31 ± 0.02 c	10.85 ± 1.08 bc	0.85 ± 0.05 ab	7.68 ± 1.79 ab	2.49 ± 0.31 ab	299.02 ± 1.55 b
	S20	1526.01 ± 6.85 b	8.33 ± 0.02 c	7.66 ± 0.91 d	0.55 ± 0.05 c	6.44 ± 1.14 b	1.73 ± 0.14 d	203.94 ± 5.46 f
	ZCK	1432.9 ± 7.52 c	8.08 ± 0.02 d	15.29 ± 0.66 a	1 ± 0.05 ab	11.4 ± 1.02 a	2.85 ± 0.11 a	296.5 ± 2.77 b
	ZW0	1675.24 ± 44.79 a	8.32 ± 0.01 c	10.96 ± 0.83 bc	0.92 ± 0.04 ab	11.25 ± 1.8 a	2.36 ± 0.25 abc	258.38 ± 0.89 c
	ZW10	1432.34 ± 10.66 c	8.69 ± 0.02 a	9.96 ± 0.39 bcd	0.96 ± 0.04 ab	9.93 ± 1.19 ab	2.26 ± 0.17 bcd	262.4 ± 1.97 c
	ZW20	1108.92 ± 18.65 e	8.54 ± 0.01 b	10.28 ± 0.73 bc	0.78 ± 0.02 abc	9.09 ± 0.52 ab	2.33 ± 0.1 abc	234.7 ± 2.71 e
Summer	CK	944.5 ± 70 ab	8.25 ± 0.13 bc	1.97 ± 0.5 bc	1.48 ± 0.37 bc	16.05 ± 4.03 bc	4.05 ± 0.52 bc	475.46 ± 96.58 bc
	S0	1429 ± 263.63 ab	8.3 ± 0.08 bc	1.85 ± 0.51 bc	1.33 ± 0.4 bc	14.53 ± 4.14 bc	3.41 ± 0.93 bc	429.98 ± 110.36 bc
	S10	1567 ± 294.35 a	8.13 ± 0.09 c	2.23 ± 0.1 b	1.63 ± 0.14 b	17.53 ± 1.31 b	4.54 ± 0.63 b	507.56 ± 46.29 b
	S20	1465.67 ± 99.68 ab	8.48 ± 0.07 ab	1.53 ± 0.57 bc	1.11 ± 0.42 bc	12.13 ± 4.55 bc	3.41 ± 1.15 bc	374.77 ± 120.75 bc
	ZCK	1481.33 ± 296.77 ab	8.37 ± 0.09 bc	1.45 ± 0.48 bc	1.06 ± 0.33 bc	11.67 ± 3.5 bc	2.8 ± 0.57 bc	363.98 ± 91.79 bc
	ZW0	1309.33 ± 353.77 ab	8.53 ± 0.02 ab	4.26 ± 0.22 a	3.21 ± 0.13 a	34.2 ± 1.65 a	8.86 ± 0.69 a	824.74 ± 49.62 a
	ZW10	804 ± 59.76 b	8.66 ± 0.03 a	0.89 ± 0.41 c	0.66 ± 0.34 c	7.37 ± 3.42 c	1.75 ± 0.81 c	206.95 ± 82.48 c
	ZW20	1365.33 ± 229.21 ab	8.39 ± 0.16 b	2.1 ± 0.37 b	1.57 ± 0.31 b	16.93 ± 3.36 bc	4.1 ± 0.62 b	483.29 ± 54.9 b
Autumn	CK	1155 ± 135.76 bc	8.46 ± 0.02 a	1.25 ± 0.56 a	0.93 ± 0.37 a	10.2 ± 4.1 a	2.52 ± 0.77 a	306.39 ± 112.46 a
	S0	1007 ± 75.03 c	8.68 ± 0.16 a	1.07 ± 0.41 a	0.78 ± 0.31 a	8.73 ± 3.32 a	1.96 ± 1.01 a	259.29 ± 107.97 a
	S10	1298 ± 47.32 bc	8.43 ± 0.1 a	1.53 ± 0.52 a	1.09 ± 0.4 a	11.83 ± 4.24 a	3.04 ± 0.97 a	356.92 ± 117.59 a
	S20	1315.33 ± 260.74 bc	8.38 ± 0.52 a	1.42 ± 0.42 a	1.04 ± 0.35 a	11.5 ± 3.73 a	2.91 ± 1.25 a	351.81 ± 107.81 a
	ZCK	1446 ± 220.01 abc	8.58 ± 0.05 a	1.73 ± 0.45 a	1.26 ± 0.31 a	13.63 ± 3.3 a	3.44 ± 1.25 a	411.05 ± 98.03 a
	ZW0	1603.33 ± 31.56 ab	8.7 ± 0.23 a	1.49 ± 0.21 a	1.09 ± 0.18 a	11.93 ± 1.88 a	2.99 ± 0.83 a	354.45 ± 55.94 a
	ZW10	1183.33 ± 278.32 bc	8.63 ± 0.16 a	1.66 ± 0.22 a	1.25 ± 0.21 a	13.4 ± 2.26 a	3.4 ± 0.81 a	402.18 ± 74.01 a
	ZW20	1831.33 ± 61.78 a	8.49 ± 0.08 a	2.46 ± 1.46 a	1.87 ± 1.13 a	20.1 ± 11.65 a	5.01 ± 2.87 a	609.18 ± 352.86 a
Winter	CK	1673.5 ± 229.81 a	8.24 ± 0.18 ab	1.4 ± 0.27 a	1.03 ± 0.19 a	11.4 ± 1.98 a	3.14 ± 0.32 a	341.98 ± 59.63 a
	S0	900.67 ± 181.6 b	8.24 ± 0.19 b	1.69 ± 0.19 a	1.25 ± 0.18 a	13.47 ± 1.86 a	3.28 ± 0.21 a	411.51 ± 57.09 a
	S10	1646.67 ± 182.74 a	8.46 ± 0.06 ab	2.83 ± 0.73 a	2.11 ± 0.53 a	22.53 ± 5.61 a	5.69 ± 1.41 a	675.98 ± 170.3 a
	S20	1401.67 ± 466.86 ab	8.53 ± 0.26 ab	1.98 ± 0.48 a	1.47 ± 0.36 a	15.93 ± 3.98 a	4.12 ± 1.2 a	488.25 ± 124.29 a
	ZCK	1309.67 ± 143.62 ab	8.36 ± 0.2 ab	2.09 ± 0.27 a	1.57 ± 0.2 a	16.9 ± 2.04 a	4.42 ± 0.51 a	515.15 ± 72.74 a
	ZW0	1148.33 ± 117.24 ab	8.58 ± 0.09 ab	2.46 ± 1.12 a	1.83 ± 0.82 a	19.57 ± 8.69 a	4.65 ± 2.14 a	589.59 ± 261.1 a
	ZW10	1762.67 ± 192.15 a	8.75 ± 0.12 a	1.34 ± 0.44 a	1 ± 0.32 a	10.87 ± 3.36 a	2.63 ± 1.03 a	336.79 ± 97.71 a
	ZW20	1751.33 ± 186.29 a	8.63 ± 0.16 ab	1.72 ± 0.75 a	1.27 ± 0.52 a	13.93 ± 5.6 a	3.2 ± 1.3 a	424.47 ± 174.63 a

Note: Different letters indicate significant differences among treatments (*p* < 0.05).

## Data Availability

The data presented in this study are available on request from the Corresponding author due to privacy or ethical restrictions.
